# Whole-genome sequencing of a *Plasmodium vivax* clinical isolate exhibits geographical characteristics and high genetic variation in China-Myanmar border area

**DOI:** 10.1186/s12864-017-3523-y

**Published:** 2017-02-06

**Authors:** Shen-Bo Chen, Yue Wang, Kokouvi Kassegne, Bin Xu, Hai-Mo Shen, Jun-Hu Chen

**Affiliations:** 1 National Institute of Parasitic Diseases, Chinese Center for Disease Control and Prevention, WHO Collaborating Centre for Tropical Diseases, National Center for International Research on Tropical Diseases, Key Laboratory of Parasite and Vector Biology Ministry of Health, Shanghai, 200025 People’s Republic of China; 2 Institute of Parasitic Diseases, Zhejiang Academy of Medical Sciences, Hangzhou, 310013 People’s Republic of China

**Keywords:** *Plasmodium vivax*, Next generation sequencing, Comparative genomics, Single nucleotide polymorphisms (SNPs), *vir*, China-Myanmar border

## Abstract

**Background:**

Currently in China, the trend of *Plasmodium vivax* cases imported from Southeast Asia was increased especially in the China-Myanmar border area. Driven by the increase in *P. vivax* cases and stronger need for vaccine and drug development, several *P. vivax* isolates genome sequencing projects are underway. However, little is known about the genetic variability in this area until now.

**Results:**

The sequencing of the first *P. vivax* isolate from China-Myanmar border area (CMB-1) generated 120 million paired-end reads. A percentage of 10.6 of the quality-evaluated reads were aligned onto 99.9% of the reference strain Sal I genome in 62-fold coverage with an average of 4.8 SNPs per kb. We present a 539-SNP marker data set for *P. vivax* that can identify different parasites from different geographic origins with high sensitivity. We also identified exceptionally high levels of genetic variability in members of multigene families such as RBP, SERA, *vir*, MSP3 and AP2. The de-novo assembly yielded a database composed of 8,409 contigs with N50 lengths of 6.6 kb and revealed 661 novel predicted genes including 78 *vir* genes, suggesting a greater functional variation in *P. vivax* from this area.

**Conclusion:**

Our result contributes to a better understanding of *P. vivax* genetic variation, and provides a fundamental basis for the geographic differentiation of vivax malaria from China-Myanmar border area using a direct sequencing approach without leukocyte depletion. This novel sequencing method can be used as an essential tool for the genomic research of *P. vivax* in the near future.

**Electronic supplementary material:**

The online version of this article (doi:10.1186/s12864-017-3523-y) contains supplementary material, which is available to authorized users.

## Background

*Plasmodium vivax* is the most widely distributed human malaria species and causes more illness than *P. falciparum* in many regions [1]. Half of the world’s population is estimated to be at risk for malaria caused by *P. vivax* [2, 3]. In China, *P. vivax* was for relatively long time the major species source of malaria infection. Due to the increasing numbers of Chinese laborers working abroad, the proportion of imported *P. vivax* was up recent years. The imported *P. vivax* malaria may bring out the high risk to the malaria-free localities where *Anopheles sinensis* mosquitoes are prevalent [4].

Previous research in *P. vivax* showed that members of multigene families are genetically variable [5]. Some mutations may help the parasite evade drug and human immune response, or stabilize the protein's structure and function [6]. This pattern was observed by sequencing *P. vivax* field isolates as well [7]. Developing novel sequencing approach directly from field samples to study genetic diversity in *P. vivax* is for a significant importance, since it allows to monitor genes involved in drug susceptibility as well as for identifying potential vaccine candidates. In *P. falciparum*, genetic diversity studies have been useful for rapidly identifying genomic regions in linkage disequilibrium leading to natural selection processes in vaccine targets and drug-resistant genes [8–11].

Driven by the increase in *P. vivax* cases and stronger need for drug development, several *P. vivax* isolates genome sequencing projects are underway and more sequence data were revealed [12, 13]. In *P. falciparum*, the genomes of many hundreds of isolates have been sequenced or genotyped already [14, 15], but the number of published *P. vivax* isolates genome is still low. Most of these projects focused on the regional characteristics of *P. vivax* isolates [16–19], and it is only recently that there have been revelations on global population genomics-based studies [20, 21]. One of the main reasons was that *P. vivax* is not amenable to continuous in vitro culture. In general, leukocyte depletion is required to minimize contamination from host. Alternatively, monkey-adapted *P. vivax* strains serve as a renewable source, but this still requires a higher cost and more steps in quality control procedures.

In this research, we sequenced and annotated the first *P. vivax* genome sequence of a clinical isolate obtained from the China-Myanmar border area (CMB-1). Genomic DNA for CMB-1 isolate was extracted from the whole blood of *P. vivax* microscopically positive patient and single *P. vivax* infection for this area was confirmed by PCR [22]. Our mapping and de-novo assembling show that this approach has similar results conformed to the method used by the past and meets the requirements of high-sensitivity mutation detection as well. It allows us to look for genetic quirks that are unique to few individuals with less expensively but greater effectiveness, particularly in the current infection circumstances.

Due to the increasing numbers of Chinese laborers working abroad, the proportion of imported *P. vivax* was up recent years. Yunnan province was still the highest transmission area in P.R. China, particularly in the southern border areas adjacent to Myanmar [23–25]. Moreover, little is known about the *P. vivax* genetic variability in CMB area. Compared to other strains and isolates, the CMB-1 isolate illustrated the highest discrepancies with the reference in principal components analysis (PCA) and could be precisely clustered according to geographic origin. Our analyses also reveal 661 novel predicted genes, suggesting a capacity for greater functional variation in *P. vivax* from this area.

The results of this study provide a novel whole-genome sequencing approach and genomic information concerning the current epidemiological scenario of vivax malaria in China-Myanmar border area, and contribute to a better understanding of *P. vivax* evolution.

## Methods

### Ethics statement

This study was conducted according to the principles expressed in the Declaration of Helsinki. After the study protocol, potential risks and potential benefits were explained to the participant, blood collection was made with written informed consent of the participant and following institutional ethical guidelines that were reviewed and approved by the ethics committee at National Institute of Parasitic Diseases, Chinese Center for Disease Control and Prevention.

### Genomic data

For our analyses, we used genome data previously published from seven monkey adapted strains: the *P. vivax* Salvador I reference strain (Sal I) [12], Salvador I re-sequenced strain [17], Belem [17], Chesson [26], Brazil-I, Peru, India-VII [5], Mauritania-I [27], and North Korean [5]. We have also referenced six human clinical isolates: Cambodia (C08, C15, and C127) and Madagascar (M08, M15, M19) [18]. We obtained the raw sequences of these strains which were deposited in the GenBank database under the following SRR number: (Sal I re-sequenced strain: SRR575089, Madagascar: SRR570031, SRR828416, SRR572651, Cambodia: SRR572648, SRR572650, SRR572649, Brazil: SRR332573, SRR332569, IQ07: SRR064844, SRR073125, India VII: SRR332913, SRR332914, North Korea: SRR332565, SRR332562, Mauritania I: SRR332413, SRR332408, Belem: SRR575087 and Chesson: SRR828528). For the India-VII, Brazil-I, North Korean and Mauritania samples, we obtained their sequences of protein coding genes from GenBank under the Assembly IDs: GCA_000320625.1, GCA_000320645.1, GCA_000320665.1, and GCA_000320685.1. In addition, we used the whole genome and CDS sequences of the Sal I reference from PlasmoDB database [28].

### Sample collection and sequencing

Genomic DNA for *P. vivax* CMB-1 sequencing was extracted from the whole blood of a *P. vivax* patient. The blood sample was collected from a symptomatic malaria-infected patient with microscopically positive returning to Tengchong county in Yunnan province in 2010. The patient is a merchant and he had been in business to Kachin state, an area of China-Myanmar border. The sample was confirmed *P. vivax* mono-species infection by *Plasmodium* species PCR-based diagnosis [23]. Genomic DNA was extracted using the QIAGEN DNeasy Blood & Tissue Kit, and sheared into 500 bp fragments using a Covaris S2 instrument. The fragmented DNA molecules were used to construct the Illumina sequencing libraries with insert sizes of 250 bp. In our previous work, we reported an initial sequencing result of this sample [22]. Our preliminary sequencing generated 31,471,932 paired-end reads and 5.86% of the quality-evaluated reads were aligned onto 96.43% of the reference strain Sal I genome in 7.84-fold coverage. Here we re-sequenced the library on Illumina HiSeq 2500 and generated 120,797,632 paired-end reads of 125 bp. All Illumina raw sequencing reads have been submitted to the NCBI Short Read Archive (SRR no. SRX1519064). We filtered all reads by removing the adapter sequences and low quality sequences using Trimmomatic-3.0 [29].

### Identification of SNP and Indel

We mapped sequencing reads from all samples to the *P. vivax* Sal I genome using BWA [30] and SAMtools-1.3 [31]. In our study the single nucleotide polymorphism (SNP) was defined as nucleotide positions covered by at least 10 reads in at least half of analyzed samples. To identify insertions and deletions (Indels), we followed the procedure described by Chan et al. [17]. Briefly, we analyzed all read pairs that did not map in the expected configuration (head-to-head within 1 kb from each other) and might be indicative of deletions or inversions.

### De-novo assembly, gene predictions and *vir* genes identification

We first removed host DNA sequences by aligning all clean paired-end reads to *Homo sapiens* genome [32] using Tophat-2.0 [33] and the non-aligned reads were considered to *P. vivax* reads. To determine the optimal k-mer, we tested different k values and compared the resulting assemblies. We then generated a de-novo assembly from the remaining corrected read pairs using SPAdes-3.5.0 [34] and a k-mer of 115 bp. Finally, contigs with no more than 500 bp length were discarded. All assembly results were submitted to the NCBI (BioSample no. SAMN03702587).

An *ab intio* gene prediction process was performed using the GlimmerHMM [35] and Augustus [36] softwares for all contigs. The original annotations of the *P. vivax* Sal I genome was downloaded from NCBI and used as the training set for the hidden Markov model. We began by comparing all partial putative genes to each other using Cd-hit [37] and discarded the shorter one when two partial genes were more than 90% identical. For the remainder partial genes, we then compared them to CDS sequences of the Sal I genome using Blast + [38]. Each partial gene with 100% identity or best reciprocal hit to reference was recognized as counterpart. The remaining genes were finally compared with the SwissProt [39] and NR (non-redundant sequence) database by Blast + (e-value cutoff of 1E-10). We discarded all partial genes without valid Blast + result and considered the rest as novel genes.

In addition, we downloaded the sequences of the whole *vir* gene family (*P. vivax* variant genes), and performed a comparison using Blast + and MEME to find all potentially *vir* genes. We then assigned each novel *vir* gene into subfamily using a phylogenetic approach in Clustal-Omega [40] to check the classification accuracy.

## Results

### Summary of the sequencing and mapping

We analyzed the genomic DNA from a malaria patient blood sample, without depletion of human leukocytes. The sequencing generated 120,797,632 paired-end reads with an average read length of 125 bp. Low-quality bases and adapter were trimmed out by using Trimmomatic-3.0. The reads were aligned to the *P. vivax* Salvador I reference strain (Sal I) genome by using BWA. An amount of 12,869,743 (10.65%) of the 120 million quality-evaluated reads were aligned onto 99.9% of the 14 chromosomes in Sal I genome. Average genome coverage was 62 times although it was variable in subtelomeric regions. Compared with other sequencing project, our result showed similar reads but with less processes (Table 1).

**Table 1:** Sequencing and mapping summary statistics for samples from field isolates and monkey-adapted strains

	Field isolates					Monkey-adapted strains	
	C08^a^	C15^a^	M08 ^a^	M19 ^a^	CMB-1	Belem^b^	Sal-1^c^
Sequencing and Mapping							
Read Pairs	231,291,984	79,414,201	215,643,747	85,703,544	*120,797,632*	81,446,663	215,743,944
Mapped on *P. vivax*	34,614,679	13,158,959	43,936,074	39,416,672	*12,869,743*	57,900,412	2,842,699
Mapped (%)	14.97	16.57	20.37	45.99	*10.65*	71.09	1.32
Mapped on Human	108,735,752	46,106,026	117,596,401	19,186,018	*98,622,782*	92,739	3,779,857
Mapped (%)	47.01	58.06	54.53	22.39	*81.64*	0.11	1.75
Coverage							
Average coverage	102	70	218	117	*62*	418	20
Genome covered (%)	93.22	93.50	95.36	97.02	*99.90*	95.73	51.43

^a^Adapted data from human clinical isolates: C08, C15, M08 and M19 [23]

^b^Adapted data from monkey adapted strains: Belem [20]

^c^Adapted data from monkey adapted strains: Salvador I [14]

**Fig. 1:**
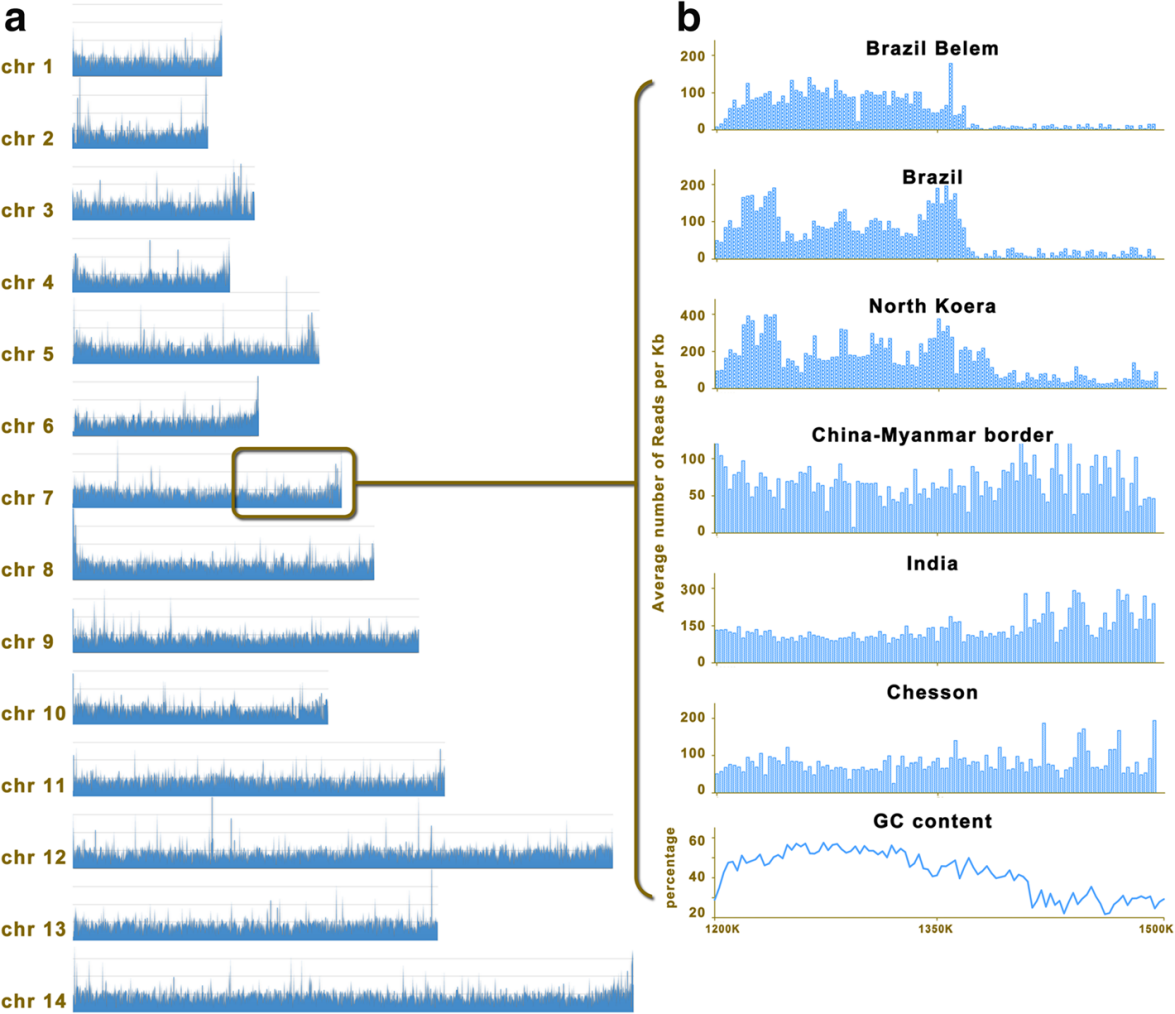
Telomeric deletion in *Plasmodium vivax* strains and isolates. **a** Pileup alignment of Illumina sequencing reads across the *P. vivax* genome. **b** A 150 kb deletion indicated by the decrease in sequence coverage (y-axis) at the telomeric end of chromosome 7. The bottom track shows the variation in GC content along this region. The lower coverage in North Korea indicates that only some of the parasites carry the deletion

A previous research has found that in South America strains (Belem and Brazil-I), very few reads could map to a 130 kb region at the subtelomeric end of chromosome 7 [7]. The main reason is a sharp decline of GC content along this subtelomeric region and accompanying enrichment of repeated sequences. In the North Korean and Cambodian samples, some parasites carried deletion while some had entire subtelomeric sequence, causing a significant but not complete reduction in coverage. However this independent deletion event did not appear in the India, Africa and CMB-1 samples (Fig. 1). It suggested that CMB-1 isolate maintained genetically homogeneous, meanwhile the subtelomeric deletion occurred in North Korea and Cambodia isolates [7].

### Sequencing shows high genetic diversity of *P. vivax* CMB-1

After the quality-evaluated reads were mapped to the *P. vivax* reference strain Sal I, 125,142 variable positions were identified. With the same method we analyzed all strain sequences onto the Sal I reference genome. Overall, we identified 108,846 nucleotide variants in at least half of the sequenced *P. vivax* strains or isolates with an average of 4.84 SNPs per kb distributed throughout the genome (Additional file 1: Table S1). Totally, 33,616 of the 125,142 SNPs were positioned in the coding region of 3,986 genes on 14 chromosomes. These SNPs caused 27,382 non-synonymous in 4,143 genes and only 6,234 synonymous mutations in 2,178 genes. We also found 4,454 of the 14,882 Indels in the coding region and 1,797 genes involved. We calculated the average SNPs per base (%) for each gene and checked the top list. Just like in a recent study [41], we found that most of these highly polymorphic genes are associated with red blood cell invasion and immune evasion such as MSP7 (PVX_082665), Pv-fam-e (PVX_089875), RBP2c, MSP1, SERA (PVX_003840), Pv-fam-b (PVX_002525), as well as VIR, such as Vir22 (PVX_097530) and Vir12 (PVX_083590). We listed in Additional file 1: Table S5, those genes with at least 5 SNPs. The SNPs from members of multigene families showed higher degree of polymorphism and caused more sequence variation, including the major merozoite invasion-realated protein family; e.g. reticulocyte binding proteins (RBPs), merozoite surface protein 3 family (MSP3s), serine-repeat antigens (SERAs), and merozoite surface protein 7 family (MSP7s) (Table 2).

**Table 2:** Classification of some important multigene families in CMB-1 isolate

Protein class description	No. of genes	SNPs per gene	Average SNPs per base (%)
reticulocyte binding protein (PvRBP)	6	70.50	0.99
Merozoite surface protein 3 (MSP3)	11	55.91	1.96
serine-repeat antigen (SERA)	13	45.46	1.53
variable surface protein (VIR)	115	30.61	1.92
transcription factor with AP2	27	20.93	0.34
protein kinase	20	18.15	0.36
Merozoite surface protein 7 (MSP7)	11	16.18	1.46
Pv-fam-d	12	12.50	0.64
DNA-directed RPB	15	10.07	0.36
PST-A	6	9.17	0.56
Phist protein (Pf-fam-b)	22	8.73	0.66
DnaJ	14	8.43	0.53
serine/threonine kinase	25	7.76	0.24
RAD protein (Pv-fam-e)	39	7.13	0.80
tryptophan-rich antigen (Pv-fam-a)	19	6.63	0.38
6-cysteine	13	6.15	0.25
all genes in whole genome	5626	5.97	0.15

Numbers are calculated for SNPs covered by at least 10 reads. “No. of genes” indicates the number of genes of a particular function class

### *Plasmodium vivax* can be distinguished by geographic distribution

**Fig. 2:**
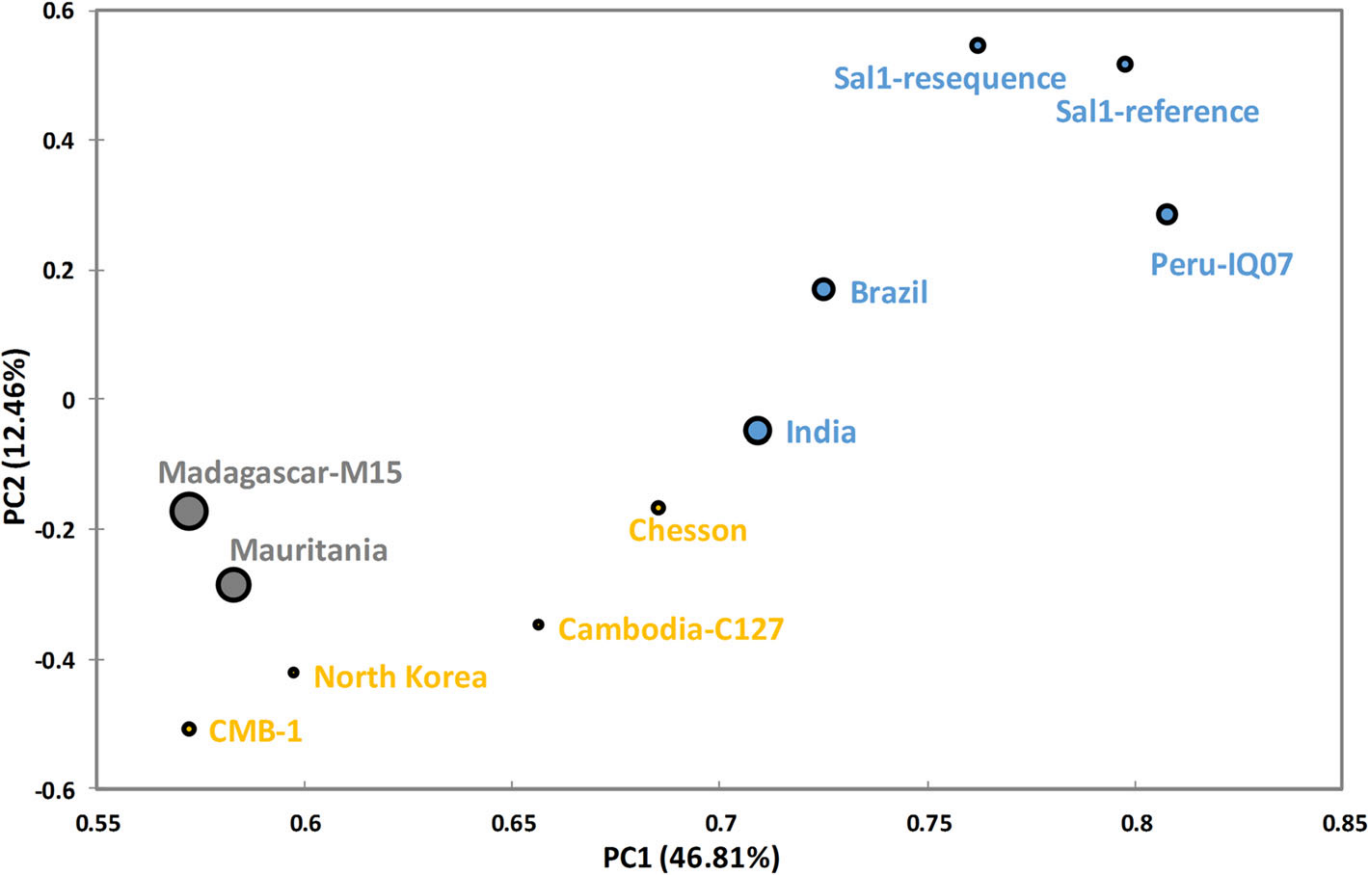
Genomic relationships among *P. vivax* strains and isolates. Principal component analysis based on 108,846 SNPs in field isolates and monkey-adapted strains. Colors correspond to the geographic origin of the samples: *blue* for Central and South America, *green* for Asia, grey for Africa and *black* for India. The PCA result showed that *P. vivax* clustered generally according to their geographic origin

**Fig. 3:**
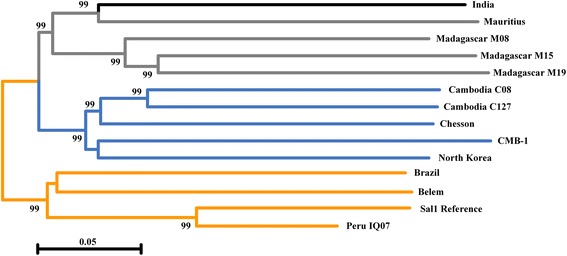
Neighbor-joining phylograms of *P. vivax* constructed from the 108,846 SNPs occurring in at least half of the samples. Lineages are colored according to geographic origin. Branch lengths indicate considerable diversity in *P. vivax* strain. Numbers at nodes indicate percentages of bootstrap support. It is worth mentioning that the India strain was tagged under the Africa category instead of Asia

To assess whether the regional differences induced genomic changes, we first performed a principal component analysis (PCA) of all strains using all identified SNPs. The PCA type is Spearman's correlation matrix, which is more appropriate on variables with different distributions than the Pearson's correlation matrix, and explained a high ratio in the first component which separates the Americas from Asia. Moreover, the reference genomes used in our PCA contained sequences from different hosts (human and monkey). *P. vivax* strains grown in monkeys serve as a renewable source of parasites, but it is unclear if these strains retain the complexity commonly observed in field isolates. Our PCA result did not reveal any clustering of samples according to their host. But, it showed that *P. vivax* clustered generally according to their geographic origin and the host switch was not a major determinant of the genetic diversity. It is similar to those of other recent studies, including one involving PCA to explore the global population structure and divides the New World from Old World samples [20]. As one of the Asia isolates, the CMB-1 isolate illustrated the highest discrepancies with the Sal I genome (Fig. 2). Thus, we constructed neighbor-joining (Fig. 3) and maximum likelihood trees (Additional file 2: Figure S1) based on the SNPs between samples with all variable positions. The phylogenetic tree was clearly clustered into three groups, including; Asia, Africa, and South America clades. As in PCA, the CMB-1 isolate looks more similar to East-Asia clades though physically located in Southeast Asia. It is worth mentioning that in our phylogenetic tree, the India strain was tagged under the Africa category instead of Asia, and more close to the South America clades in PCA approach.

**Fig. 4:**
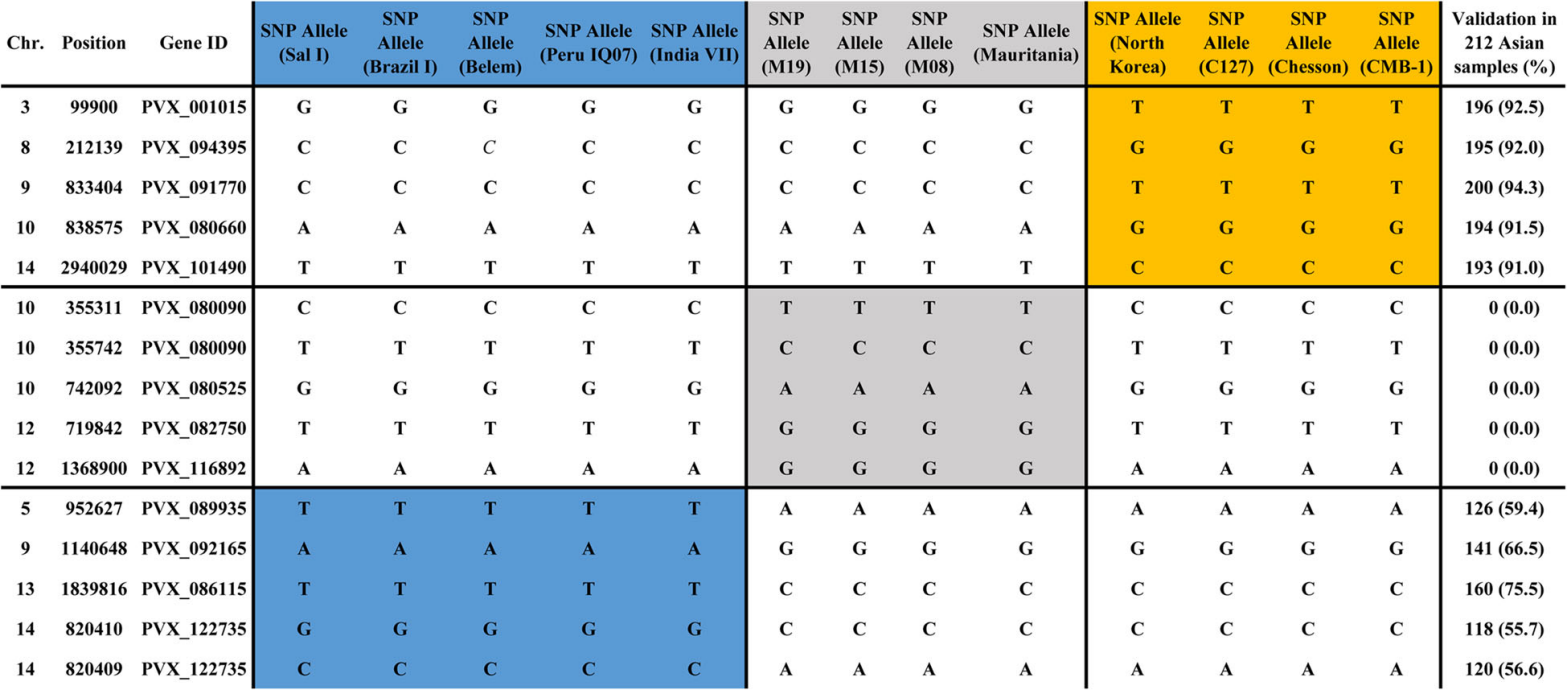
Example with 15 SNPs provides baseline to distinguish parasite infections and their geographic origins. The positions of the SNPs are shown along with the chromosome on which the SNP resides, and the position (coordinate number from PlasmoDB) on that chromosome, the Gene ID where the indicated SNP is located, and the reference and alternate alleles

In order to provide a standardized genetic marker set that identifies a genomic signature, Baniecki et al. [42] have defined a barcode consisting of 42 SNPs and analyzed the performance on 87 *P. vivax* clinical samples in South America (Brazil, French Guiana), Africa (Ethiopia) and Asia (Sri Lanka). Our results confirmed the existence of the 42-SNP barcode as a marker that identifies genomic signatures. However, as shown in Additional file 1: Table S2, the SNPs marker was less effective when we expanded the scope of testing to other strains or isolates (CMB-1, Belem, Chesson and Mauritania). In our study, the SNPs were divided into 3 geographically group and 539 of the 125,142 variable positions showed the consistency of the geographic distribution and were independently informative. We validated our markers in a SNP dataset released recently [21], 480 of the 539 SNPs could be found. Within the 212 Asian samples, we found 2,750 loci in 130 African unique SNP markers (2,750/27,560) and 41,005 loci in 275 Asian markers (41,005/58,300). These SNP markers are very effective in distinguishing the African and Asian samples. However we also found 12,984 loci in 75 mixed region marker (12,984/15,900), suggesting that the existing data do not yet support the New World samples signature. As shown in Additional file 1: Table S3 and Fig. 4, these SNPs marker listed for each group were various and involved in different genes.

### *De novo* genome assembly of *P. vivax* CMB-1

We removed DNA sequences originating from the host genome by filtering the sequences similarity to *Homo sapiens* genome. The remaining reads were *de novo* assembled. We yielded a database composed of 8,409 contigs, with an average GC content of 39% and N50 lengths of 6.6 kb (Additional file 1: Table S4). A total of 29,755,552 bp of the estimated genome length was assembled with 5,714 contigs larger than 1,000 bp. We mapped all de-novo assembled CMB-1 contigs to the *P. vivax* Sal I genome. There are 5,966 of 8,409 contigs mapped to 14 chromosomes of Sal I genome using Blast + accounting for 22.5 Mb (or 99.8% of the 14 chromosomes overall length) with identity up to 95%. We then applied a mixed gene prediction process to identify putative genes in all of the contigs and compared the predicted genes with CDS of Sal I genome. A total of 16,028 partial genes loci was predicted by the ab intio gene prediction process. A number of 9,985 partial genes were high sequence similarity recognized as whole or exon part of all the 5,614 genes of *P. vivax* Sal I. Among them, 5,382 partial genes were discarded as valid Blast + results on common database were lacked. Overall, we identified 661 novel *P. vivax* genes, among which 258 genes were similar to annotated *Plasmodium* hypothetical proteins and 32 genes were similar to well-characterized *Plasmodium* genes.

In a recent research by Cornejo et al.*,* authors found that natural selection acts not only by shaping the patterns of variation within genes but it also affects genome organization [41]. The problem of polymorphism and number of paralogs affect gene families differently. In this study we checked the SERA and MSP3 families to see whether any different patterns exist. The Sal I reference genome contains 12 MSP3 and 13 SERA genes. A total of 35 partial genes mapped to them with high identity but only 20 could be recognized as their orthologous by best bilateral Blast + contrast. The lack of 4 MSP3 (PVX_097685, PVX_097700, PVX_097715 and PVX_097695) and 1 SERA (PVX_003840) genes in CMB-1 isolate suggested a higher polymorphism which came from recent duplication events within the *P. vivax* lineage [43, 44].

### Identification of novel *vir* multigene family member

**Fig. 5:**
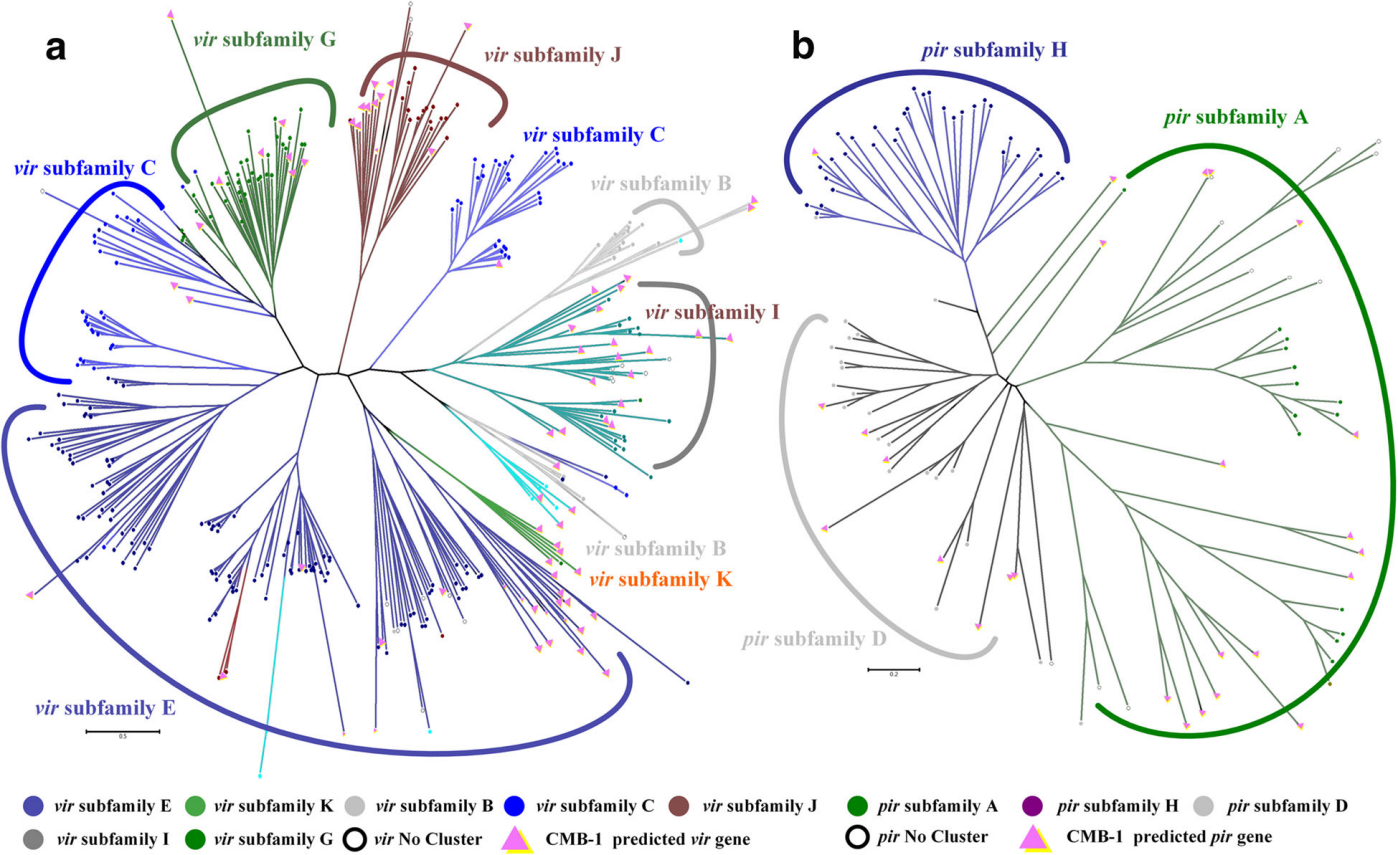
Analysis of novel *vir* and *pir* genes family predicted from the CMB-1 isolate. **a** Phylogenetic tree showing the relationships between the protein coding sequences of *vir* genes from the Sal I genome (dots) and those predicted from the CMB-1 contigs (triangle). Annotated *vir* genes (dots) are colored according to their subfamilies. Nodes used to assign predicted CMB-1 *vir* genes into subfamilies are shown by the colored branches derived from them. **b** The relationships between *pir* genes from the Sal I genome (dots) and those predicted from the CMB-1 contigs (triangle)

**Fig. 6:**
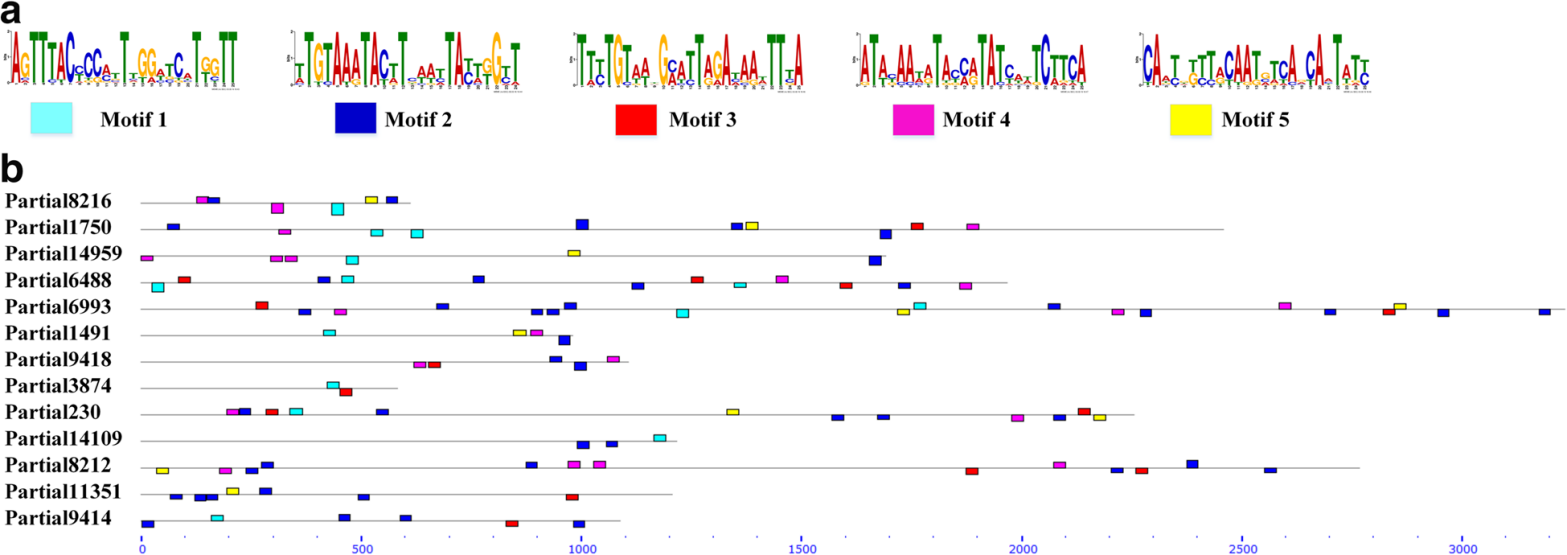
An example of the motifs and organization in novel *vir* gene (subfamily J). **a** Five most conservative motifs derived from the published *vir* genes (subfamily J). **b** List of our novel *vir* genes in subfamily J, horizontal lines illustrate the number of domains, their architecture and approximate location in the gene sequence. The different-colored squares show the corresponding motif in part A

The *vir* superfamily is variably expressed and encodes proteins that are exported to the host cell surface for the purpose of evading the host adaptive immune response [45]. The revealed *P. vivax* Sal I genome divided 346 *vir* genes into 12 different subfamilies. Later, Francisco et al. [46] confirmed that subfamilies A, D and H cannot longer be classified as *vir* genes. From 165 *vir*-like novel genes we identified 78 *vir* genes and 26 *pir* genes (Fig. 5). We then assigned each novel *vir* gene into corresponding subfamily using Clustal-Omega and reconstructed a phylogenetic tree using MEGA6 [47]. For each subfamily, we identified the most conservative motifs on the published *vir* genes in each subfamilies using MEME [48], and confirmed that these motifs exist in our novel genes (Fig. 6). Our analyses revealed that the proportion of novel *vir* genes assigned to subfamilies were quite different and suggested the presence of different subfamilies in different samples due to rapid accumulation of mutations. Overall, our analysis reinforces the notion that *vir* genes are extremely diverse, and the current catalogue of *vir* genes is likely to be far from complete.

## Discussion

One of our main purposes of this study was to determine whether our simplified sequencing approach caused systematic errors in genomic level. Sequencing the *P. vivax* genome has provided us insights into parasite biology but also has arisen many challenging questions. The major bottleneck for *P. vivax* studies is the fact that the parasite is not amenable to continuous in vitro culture. In general, the *P. vivax* DNA was extracted directly from patient blood samples with leukocyte depletion to minimize contamination from host. However, it always raises the question that the parasitemia is typically less than 10,000 parasites per μl of blood [17]. Furthermore, the parasites obtained from blood samples of infected patients are contaminated by the large proportion of human genomic DNA. This leads to a large consumption of blood sample in sequencing.

Therefore as an alternative, monkey-adapted *P. vivax* strains are typically generated by direct injection of parasitized erythrocytes from patients, and serve as a renewable source of parasites for in vitro and ex vivo experimental studies. Even though some studies have shown that monkey-adapted strain genomes remain representative of the original parasite genomes [7], and this approach still need more steps in quality control procedures. For example in Belem monkey-adapted strain, it still retains 0.11% human DNA contamination (Table 1).

Previous studies [17] have shown that a variable proportion of reads (15–46%) from field isolates could be mapped to the *P. vivax* genome and provided enough coverage. Judging from coverage, the monkey-adapted strains effect even better (Table 1). The Belem monkey-adapted strain showed more coverage in less mapping reads due to its large volume of sample and easy to remove leukocytes. However, the average genome coverage does not accurately represent the quality of the sequencing data. By contrast, only 10% of our CMB-1 sample reads mapped to the *P. vivax* genome with minimum coverage but provided the highest genome covered ratio. Furthermore, a great deal (22–58%) of the reads generated from field isolates mapped to the human genome although the blood samples were already processed on CF11 columns. This means that host DNA cannot be completely erased (Table 1).

Overall, our analyses suggested that direct sequencing approach requires only high parasitemia for vivax samples without leukocytes filtration, and produces more available reads with less processes (Table 1). By reducing manual steps, it both reduces errors and time, and is also cost-effective. The direct sequencing approach will be an essential tool for the study of this important malaria parasite.

The increasing Chinese investment and numbers of laborers working abroad bring out high risks for malaria infection from endemic areas. Myanmar was still the main imported source of vivax malaria and the number of patients from this area was increased significantly. Previous studies uncovered high degree of genetic polymorphism in *P. vivax*, which was translated into functional variation [5]. Our sequencing analysis identified high levels of genetic variability in members of multigene families in CMB-1 isolate. The SNP rates averaged 8.11 per gene in mutational genes and 5.97 per gene in whole genome (Table 2). In contrast, some multigene families such as reticulocyte binding proteins (PvRBP), merozoite surface protein 3 family (MSP3), serine-repeat antigens (SERA), variable surface proteins (*vir*) and merozoite surface protein 7 family (MSP7) had SNPs rate of 70.50, 55.91, 45.46, 30.61 and 16.18 per gene, respectively. Some members of these protein families have been shown high immunoreactive previously [49, 50]. We also observed the highest enormous diversity in MSP3 and *vir* genes (1.96% and 1.92% SNPs per base, respectively), far greater than any other family. Our findings confirm previous researches indicating that members of multigene families are high genetically variable [19]. On the other hand, the high number of SNPs in gene family is one of the common manifestations of genetic complexity, which usually comes from high genetic polymorphism. The polymorphism and number of paralogs affect gene families differently in *P. vivax* [41], and easily impede short reads accurate mapping. These conserved regions of gene family paralogs are similar but not identical and always lead to a high score with low quality alignment, especially in *P. vivax* which exhibit extraordinary genetic diversities. More studies in CMB area are still needed to further refine our estimates.

Our mapping approach shows that 22,174,850 (18.36%) CMB-1 reads cannot map to host genome, but at the same time only 10.65% reads can be mapped to *P. vivax* reference. These remaining ~8% reads should also be considered as a part of CMB-1 genome and effectively increased nucleotide coverage in de novo assemble processes. The same situation also occurred in the field isolates where numerous proportions of reads (25–38%) mapped neither host nor *P. vivax* reference genome.

By de novo assembly, we also identified 661 novel predicted genes, including 78 *vir* genes and 26 *pir* genes. We observed that these novel *vir* genes predicted in the CMB-1 isolate shared the same motif with given *vir* genes, regardless of whether they are located in reference genome or in some novel contigs which do not match the reference genome. The conserved structure suggests that most of the novel *vir* genes might be functional. In another hand, we found enormous variations in *vir* genes sequence from the same subfamily and the proportion of genes assigned to subfamilies was quite different between Sal I and CMB-1. This reinforces the notion that *vir* genes are extremely diverse between *P. vivax* strains [26].

In this study, we also present a 539-SNP data set for *P. vivax* that spanned all 14 chromosomes of the genome and involved 267 genes. Studies on SNP barcode have an early start, but most of them pursued higher adaptability of the classification and lower sample cost. Here we have given more attention to increase the accuracy and detect genotypes with high sensitivity. As shown in Additional file 1: Table S3, our SNP markers provide an informative data set to identify different parasites from Asia and Africa. The whole genome SNPs distribution data will help us further to identify the *P. vivax* infections from China-Myanmar border area.

The findings showed in this paper provide the whole genomic information of a vivax malaria case in China-Myanmar border area, where little is known about the genetic variability. The results of this work contribute to the acquisition of some knowledge on *P. vivax* genetic variation, especially for multigene families, from China-Myanmar border area.

## Conclusions

Here we report the first *P. vivax* isolate (CMB-1) genome sequence of a clinical isolate in this area using a direct sequencing approach without leukocyte depletion. We present a 539-SNP marker data set for *P. vivax* that can identify different parasites from different geographic origins, and identified exceptionally high levels of genetic variability in members of multigene families. We also found that the direct sequencing approach could produce more available reads for mutation detection, and be used as an essential tool in the near future.

## Additional files

Additional file 1: Table S1-S5.
**Table S1.** 33,616 of 125,142 SNPs in the coding region of 4,145 genes on 14 chromosomes. **Table S2.** 42-SNP barcode marker that identifies genomic signatures. **Table S3.** 539 of the 125,142 variable positions showed the consistency of the geographic distribution. **Table S4.** De novo assembly summary statistics of the *Plasmodium vivax* CMB-1 genome. **Table S5.** Average SNPs per base (%) of 1,826 genes with at least 5 SNPs. (XLSX 2120 kb)
Additional file 2: Figure S1. Maximum-Likelihood phylogenetic tree of *P. vivax* constructed from the 108,846 SNPs occurring in at least half of the samples. Lineages are colored according to geographic origin. Branch lengths indicate considerable diversity in *P. vivax* strain. Numbers at nodes indicate percentages of bootstrap support. (TIF 254 kb)

## Abbreviations

CMB: China-Myanmar border area
MSP3: merozoite surface protein 3
PCA: principal components analysis
PCR: polymerase chain reaction
RBP: reticulocyte binding protein
SERA: serine-repeat antigen
SNP: single nucleotide polymorphism
*vir*: variable surface protein
