# Supplementary figures and images for: Whole-genome sequencing of a Plasmodium vivax clinical isolate exhibits geographical characteristics and high genetic variation in China-Myanmar border area
Source: BMC Genomics. 2017 Feb 6;18:131. doi: 10.1186/s12864-017-3523-y (PMC5294834; doi:10.1186/s12864-017-3523-y)

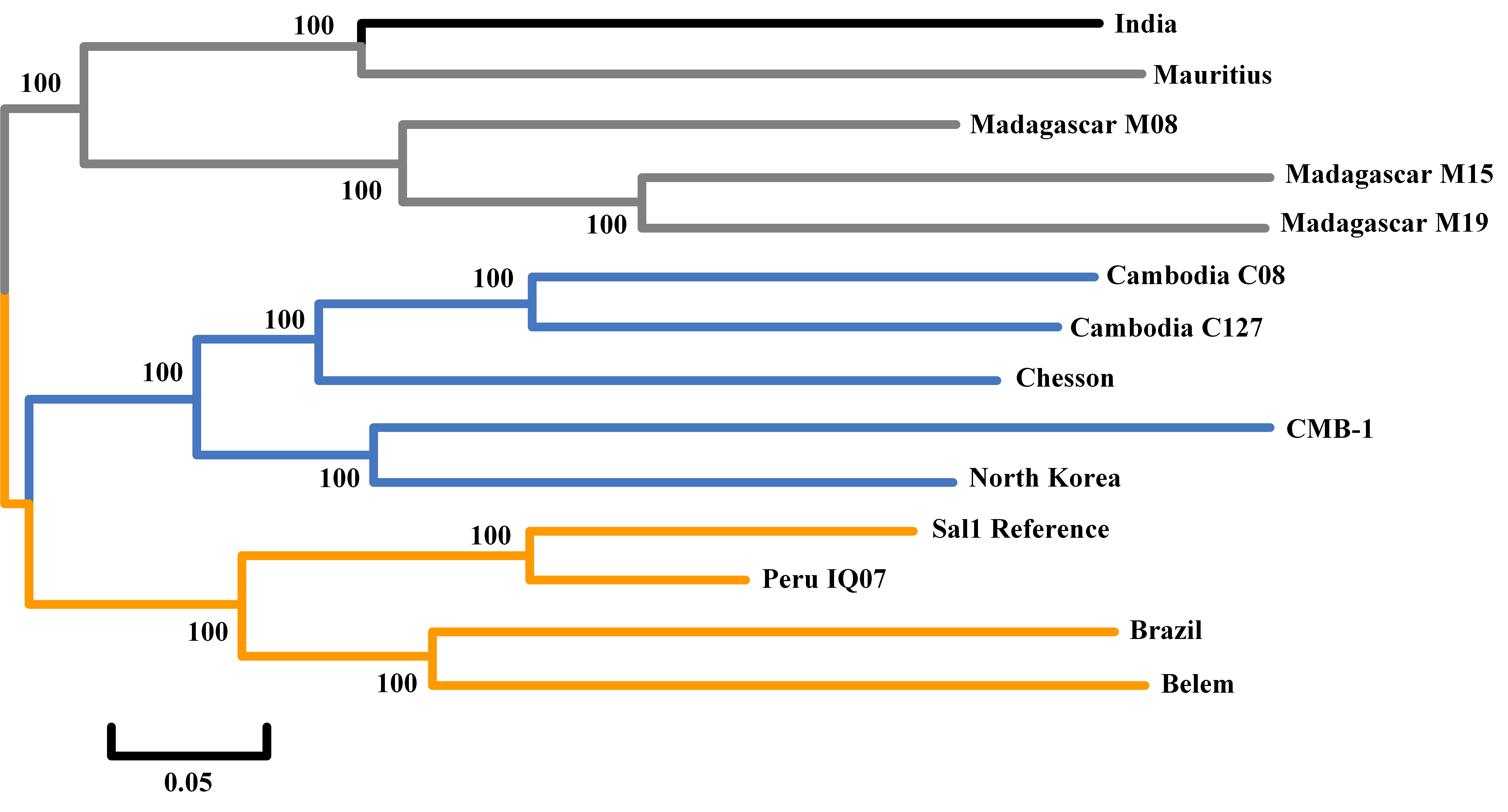

Supplement: Additional file 2: Figure S1. — Maximum-Likelihood phylogenetic tree of P. vivax constructed from the 108,846 SNPs occurring in at least half of the samples. Lineages are colored according to geographic origin. Branch lengths indicate considerable diversity in P. vivax strain. Numbers at nodes indicate percentages of bootstrap support. (TIF 254 kb) [file 12864_2017_3523_MOESM2_ESM.tif]
